# Bilateral polyotia: case of a rare and atypical congenital malformation in a three-month-old infant

**DOI:** 10.11604/pamj.2021.38.63.27458

**Published:** 2021-01-20

**Authors:** Roger Christian Meva’a Biouélé, Emmanuel Choffor-Nchinda

**Affiliations:** 1 Ear, Nose and Throat (ENT) Unit, Yaoundé Central Hospital, Yaoundé, Cameroon,; 2 Department of Ophtalmology, Ear, Nose and Throat (ENT) and Stomatology, Faculty of Medicine and Biomedical Sciences, University of Yaoundé I, Yaoundé, Cameroon,; 3 Ear, Nose and Throat (ENT) Unit, Buea Regional Hospital, Buea, Cameroon,; 4 Department of Surgery and Specialties, Faculty of Health Sciences, University of Buea, Buea, Cameroon

**Keywords:** Polyotia, congenital malformation, plastic surgery, Cameroon

## Image in medicine

Polyotia is an extremely rare congenital malformation of the external ear, characterised by the development of an accessory auricle. It is more often unilateral. We report the case of a Cameroonian infant whom we received at the age of three months. The infant's mother had noticed a deformity concerning both ears since birth and did not observe any other associated symptom. On physical examination, we found an additional auricle fusing with the tragus on both sides. This unusual structure consisted of skin and cartilage. Their greater diameters measured 2.5cm and 2cm on the left and right respectively. Otoscopic evaluation and general physical examination were normal. A computerized tomography (CT)-scan of the temporal bones as well as an abdominal ultrasound scan were requested to explore other possibly associated anomalies. These imaging studies were normal. The diagnosis of isolated bilateral polyotia was made. An ear reconstructive surgery was proposed to the parents. An informed consent was obtained from the parents, pre-operative work-up was requested and the procedure was planned. A bilateral otoplasty was done under general anaesthesia. The procedure was simple, consisting of excision of the excess tissue to give a natural aesthetic result. Post-operative evolution was favourable, with good scarring on day 10, as illustrated in the figure. Nine months following the procedure, the parents seemed satisfied with the aesthetic result. Polytia is a rare congenital malformation. Management requires adequate and comprehensive investigations to exclude enchondroma or associated malformations, prior to ear reconstructive surgery.

**Figure 1:**
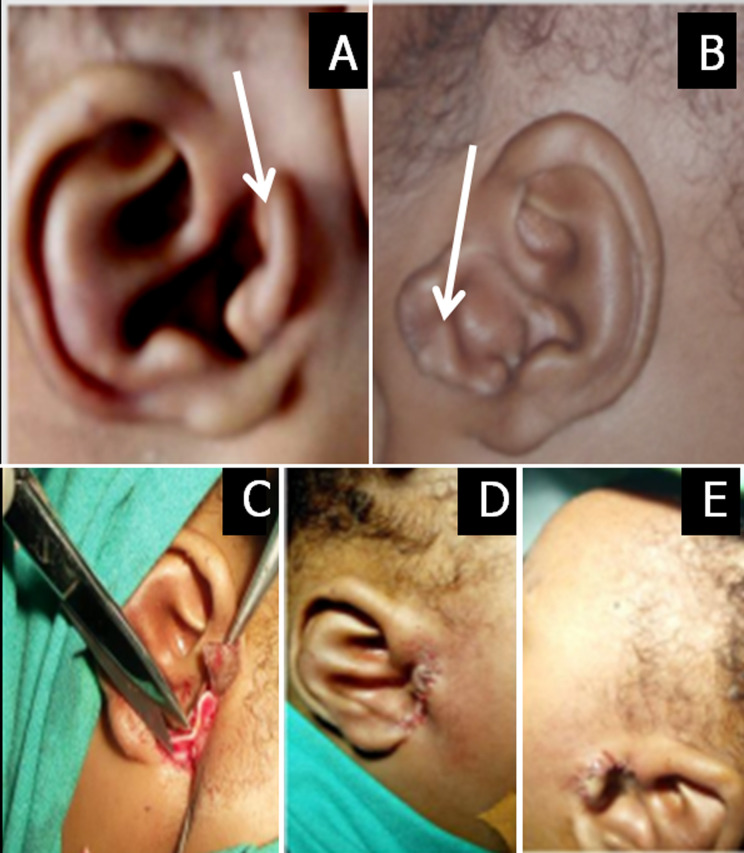
accessory pinna fusing with the tragus; A) right ear; B) left ear; C) operative view showing excision of excess cartilage and skin; D) postoperative aspect of auricle after 10 days; (E) postoperative aspect of auricle after nine months

